# Ethics Committees' Practices in Healthcare, Banking and Research: Key Requirements for Their Functionality

**DOI:** 10.1111/jep.14310

**Published:** 2025-01-27

**Authors:** Tuğba Arık, Susanne Michl

**Affiliations:** ^1^ Berlin Institute of Health Charité‐Universitätsmedizin Berlin Berlin Germany; ^2^ Institute of the History of Medicine and Ethics in Medicine Charité‐Universitätsmedizin Berlin Berlin Germany

**Keywords:** business ethics, code of ethics, decision making, dual use of research, ethics committees, medical ethics, organizational ethics

## Abstract

**Rationale:**

To meet concerns about ethical and unethical behavior in their work environments and workplaces, organizations began establishing ethics programs that contain ethics committees (ECs). There is now a tradition and diverse use of ECs for ethical decision‐making in many different organizational settings. In addition, ECs have been subject to many publications in books and articles in the scientific literature. Yet, until now no comparative analysis has been published that brings together ECs' practices in different sectors.

**Aims and Objectives:**

This article aims to bridge this knowledge gap and illustrate which main requirements for ECs' practices need to be addressed to help ECs meet their anticipated functions.

**Method:**

To do so, this paper lays out a study based on an exploratory, qualitative design using focus groups and individual expert interviews that compare ECs' practices in the healthcare, banking, and scientific research sectors (as far as dual use of research is concerned).

**Results:**

Based on the results of this study we were able to make a distinction between two main categories: *moral authority* and *trustworthiness*. We were also able to identify three sub‐categories: *legitimation*, *mode*, and *outreach*.

**Conclusion:**

Based on the exploratory analysis in this study, we conclude that there are the following three distinct main requirements for the functionality of ECs: (1) a dialog between EC members and other stakeholders, (2) an approach that considers various possible modes (reactive, screening, moderating, and preventive) to enhance the quality of ECs’ decision‐making processes and (3) an outreach to all relevant EC stakeholders for the further validation of the main requirements found for ECs functionality.

## 1 Introduction

While issuing ethics in the healthcare and scientific research sectors enjoys a long tradition (e.g., [1, 2]), the relevance of ethics for organizations operating in the banking sector became apparent at the latest with the collapse of the financial system in 2007–2008. The height of the Covid pandemic from 2020 to 2023 has also shown that crises can come suddenly and may not necessarily represent an exception. Instead, these crises confirmed the importance of times when urgent decisions needed to be made. This illustrates the importance for organizations to develop their own mechanisms for ethical decision‐making in their work environments and workplaces (e.g., [3, 4, 5, 6, 7]). One answer to meet these demands can be that organizations establish ethics programs (e.g., [1, 8, 9, 10, 11, 12]). According to [10] ethics programs can be described as “the formal organizational control system designed to impede unethical behavior”. These ethics programs usually consist of the following components: a code of ethics,^1^ ethics hotlines^2^ and/or an ethics committee (abbreviated to EC) [8, 9, 10].

A study conducted by Kaptein [10], based on survey respondents' self‐assessment of observed unethical behaviors, indicates that in banking, the more the ethics program components are used, the more the occurrence of unethical behavior is reduced. This reduction identifies the quantity of ethics program components as one potential factor that can enhance ethics in an organization. While codes of ethics and ethics hotlines have received much scholarly attention in the scientific community dealing with business ethics [10, 13], ECs have received less attention. Presumably as a consequence of this, it is not self‐evident that every business organization makes use of an EC. However, today ECs seem to be in an even more prominent position in banking than they have been in their original context, the healthcare sector (e.g., [1, 14, 15]).

ECs exist in diverse organizational settings and sectors (Verwij, Brom, & Huibers, 2000). For example, in organizational practice, we know of ECs operating in the healthcare sector, such as the clinical ECs, animal ECs, and research ECs. There are also ECs operating in governmental agencies such as ECs of government councils or ethics commissions [1, 16]. In practice, we can also observe that even a decade ago almost every second business organization also has installed an EC, as shown by Callaghan, Wood, Payan, Singh, and Svensson [17]. Out of the top Fortune 500 corporations in each country, a large percentage had ECs. In Canada, it was 55.9%, in Australia 42.1%, and in the US, even 74.4% of the top 500 companies have an EC.^3^ So far there is not an up to date, single, overarching study existing that provides an overview about the prevalence of ECs in all types of organizations and sectors.

We have focused in this study on ECs in the healthcare, banking, and scientific research sectors that operate on a non‐mandatory basis. ECs in these three sectors belong to different EC generations. Those in the healthcare sector have a long tradition of dealing with ethical questions or challenges (e.g., [1]). Those in the banking sector were introduced to ethical question resolution in the last two decades.

The research ECs in this study are those with a focus on dual use research of concern.

The term “dual use research of concern” describes research that is intended to provide a clear benefit, but which could easily be misapplied to do harm, according to the World Health Organization and the joint recommendations of the German Research Foundation (DFG) and the German National Academy of Sciences Leopoldina ([18]; www.leopoldina.org). Although efforts to address health security involving biological and chemical risks are not new, ECs that explicitly address dual‐use research of concern emerged after 2011 (e.g., [18, 19, 20]). Therefore, these ECs can be described as belonging to the youngest generation that is currently emerging and just beginning to create standards to address ethical issues (e.g., [18, 19, 21]).

We excluded explicitly those research ECs with a legally mandatory framework like ECs specifically for preclinical and clinical studies. These legislatively constrained ECs are likely to operate on differently institutionalized structures having a more regulating or controlling character. The three EC types of our study are thus based on ECs with non‐mandatory purposes that have a more advice‐giving character.

It is surprising that despite this tradition and diverse use of ECs in organizations, no investigation to date has been conducted that compares institutionalized practices of ECs operating in different sectors and that identifies main requirements for their functionality. Although, we believe that each sector could benefit from this comparison, since, for example among others, a solution strategy used by ECs in one sector could be of interest to ECs in another sector. Also, since EC activities across sectors can be considered as similar at least to some extent, there may be overarching guidelines and training materials that could be shared among ECs in different sectors, meaning that not each EC needs to develop these resources on its own. This paper aims to meet this knowledge gap, at least partially, in the scientific literature. We do so by taking a very first step for orientation when it comes to comparing between ECs in these three sectors, by identifying main requirements or obstacles that organizations need to tackle to install ECs that will operate well in covering their anticipated functions.

Our study is based on an explorative, comparative design using the qualitative research methods of focus group and individual expert interviews. The overarching research question that guided this study was the following:

“Based on a comparative, cross‐sectoral approach to investigate ethics committees, what are the main requirements for the functionality of ethics committees?”

The inductive research approach used allowed us to generate hypotheses about requirements and to reflect on our findings to set an agenda for further validation of the found main requirements for the functionality of ECs.

## 2 A Definition, the Roots and Institutionalization of Ethics Committees

### 2.1 Towards a Definition of Ethics Committees

In the scientific literature there are many descriptions of ECs. For example [1], describe ECs as “a multidisciplinary body that meets … to address ethical issues that arise in the … organizational setting.” According to [22] ECs “are multidisciplinary committees established to address ethical dilemmas in patient care”. Others such as [15] define ECs as “a group of persons that, on an optional or mandatory basis, advise, discuss, or consult … about ethical issues …” [23] define ECs as “a formally specified group of employees responsible for developing, updating, and enforcing the code of ethics.” Considering these definitions of ECs, it might become clear that ECs can exist in different organizational settings and that they can serve different functions within these organizational settings.

Indeed, according to further scientific findings, ECs are found in diverse contextual settings in the society [1, 16]. There are clinical ECs, research ECs (such as institutional review boards, dual use research of concern ECs or animal research ECs), ECs of government councils, ECs in federations of healthcare institutions, ECs in professional associations, national and institutional discussion platforms for science and bioethics and ECs in business organizations [16].

In this study we focus on ECs operating in the healthcare, banking, and scientific research sectors. Furthermore, for the purpose of this comparative study we use a broad definition for ECs put forward by [24]: “An EC can be defined as a group of people who are appointed to address ethical issues [by an organization].” However, bearing in mind the ECs in these three sectors that we are focusing on in this study, we can state that the specific ethical questions addressed in these ECs might differ. For example, ECs in the clinical sector foremostly focus on ethical questions such as ethical challenges in reproduction, in the care of disabled persons, or end‐of‐life issues on a more micro level (e.g., [1]). Prototypical ethical questions put forward in this frame can be found in Box 1.

BOX 1 **Examples for ethical questions in the healthcare sector.**

- Is it ethically right to treat a patient against their will when the choice made by that patient to refuse treatment has dire consequences (disability, death)?
- When is it ethically right to limit or stop patient treatment plans?
- Does a patient understand the information given on benefits and risks of treatment and does the patient give consent?
- If a patient is mentally incompetent to communicate their preferences and the preferences before the treatment are unknown, who is the appropriate contact person to approve that treatment?
- Is it ethically right to forgo life‐sustaining treatment(s)?
- What is ethically right when problems in the allocation of scarce resources arise?
- What is ethically right when conflicts of interest can arise, for example: does the physician benefit financially, professionally by ordering specific test, prescribing medications, seeking consultation etc. or do, for example, religious beliefs lead to conflicts of interest in the decision‐making for a treatment?

13

ECs in the banking sector tend to focus not only on ethical issues at the individual, micro level, such as how to deal with vulnerable clients, for example those suffering from dementia or other limitations, but also on broader organizational or societal issues at the meso and macro levels. These broader issues might include deciding when to exclude certain clients who cause social harm, such as those who work with the arms industry, which causes harm to human rights and potentially to human life. Another example would be carefully considering the long‐term ethical implications of a particular product before introducing it. Prototypical ethical questions put forward in this frame can be found in Box 2.

BOX 2 **Examples for ethical questions in the banking sector.**

- What is the right ethical decision to be made for a bank as an internationally operating agricultural bank when, for example, the rise of meat consumption in one country causes rampant deforestation in another country?
- Is it appropriate to charge a business a higher risk margin if data leads the bank to believe that its performance is lagging by its own doing (e.g., the business owner)?
- How to deal in an ethically right manner with clients that lose their self‐efficiency?
- Is it appropriate to make a relevant offer to a client who is experiencing an important life event such as becoming a parent?
- Is it appropriate to share data with third parties, for instance with the seller or with the seller's real estate agency when a client buys a home?

14

The overarching ethical question that guides discussions in ECs focused on dual use research of concern is to prevent the use of harmful outcomes that potentially can evolve from materials, knowledge, and technologies based on research and innovations. These can arise in the biological field, but also in other disciplines (e.g., [21, 27]). Prototypical ethical challenges that are discussed in these ECs are summarized in Box 3.

BOX 3 **Examples for ethical questions in the scientific research sector.**

- Could the materials/methods/technologies or knowledge of concern that are used in organizations in the scientific research sector, physically or in any other way harm people, animals or the environment, by themselves or if modified or enhanced?
- Could the materials/methods/technologies or knowledge concerned that are used in organizations in the scientific research sector, physically or in any other way, have direct negative impacts on the security of individuals, groups or states?
- Does the activity used involve the development of surveillance technologies? What would happen if they ended up in the wrong hands?
- Does the (research) activity generate knowledge, materials, and technologies that could be used for criminal or terrorist purposes?
- Could the activity result in the development of chemical, biological, radiological or nuclear (CBRN) weapons or any method for their delivery?

15

### 2.2 The Roots and Institutionalization of Ethics Committees

The time periods for the emergence and evolution of ECs in the healthcare, banking and scientific research fields differ from one another. However, one common element that ECs in these sectors share is that they among other evolved as a reaction to a larger social transformation in which one hallmark was the heightened notion of human rights and similar ethical concerns [1, 20, 21, 24]. In these sectors ethical, legal, scientific developments and/or developments in business practice created potentially a higher interest to critically reflect on the process of how for example scientific information is translated into an organization's practices. These practices could be in the medical or business community, and such developments often spurred the medical or business community to change their practices in a responsible manner as a reaction to organizational scandals. That is where ethics committees entered the scene.

Overall, it can be said that the origins of ECs in an organizational context can be traced back to the clinical setting in the US (e.g., [14, 15]). For example, in the 1920s the sterilization legislation, which was a result of the eugenics movement, led to the formation of committees to regulate clinical decisions. These sterilization committees, mainly consisting of physicians with experience in the management of humans with a mental disability during an age of few if any human rights for these subjects, made “objective” decisions to avoid the social burdens of inherited disabilities (e.g., [14, 15, 28]).

Less prejudicial methods were established in the form of clinical ECs that have at least the following four ancestors:

1. Dialysis patient selection committees,^4^ that arose in the 1960s after long‐term dialysis became available, although costly.
2. Prognosis committees, that were significantly influenced by the well‐known 1976 New Jersey case of Karen Ann Quinlan.^5^
3. Abortion selection committees, which were used until the 1970s in the United States and mostly identified pregnancy terminations due to medical or psychiatric conditions [14].
4. Institutional review boards (IRBS) that were charged with reviewing all publicly funded research according to ethical standards [1, 29].

Nowadays, almost all larger hospitals in the US have established ECs, both clinical ECs and IRBs (e.g., [30]).

A comparable evolution of clinical ECs in the European context took place beginning in the Netherlands and soon after spread over to Germany (e.g., [29, 31]). As recounted by Woellert [29], the heightened awareness of ethical questions in healthcare arose in the 1970s, mainly influenced by individuals who maintained professional relationships with practitioners of clinical ethics in the US. This led in the 1980s to the establishment of relevant platforms for professional exchange such as the German Academy of Ethics in Medicine (AEM).

In the subsequent decades, ethics structures in German healthcare organizations were decisively influenced by a position paper published by the two Christian Hospital Associations in 1997 that recommended the establishment of ECs in hospitals with a Christian sponsorship. Hospitals with other funding later followed these examples. Around 2000 in Germany, it became commonly accepted in debates among clinical ethics experts to talk about organizational ethics that would incorporate all organizational relevant aspects that can have an influence on the ethical quality of patient care.

Today the integration of ECs into healthcare organizations is being implemented and not just recommended by relevant players in the sector like the German Federal Chamber of Physicians and the German Ethics Council (Deutscher Ethikrat). This implementation comes from ethics structures and ECs being put on top management agenda in German healthcare organizations [29]. Although, the number of German hospitals installing an EC is increasing continuously, local differences in the structures and resources for ECs do exist (e.g., [32]).

In contrast to the institutionalization of ECs in the clinical setting, the idea of using ECs in companies, and with this in the banking sector, can be regarded as a new development. As mentioned according to Arik et al. [24] the rise of ECs in companies occurred during a broader ethics reform due to corporate scandals such as those of Enron and Tyco, at the start of the century. Only two decades later, the tide had turned and many companies worldwide as mentioned above had installed ECs.

In the European setting the installation of ECs is especially observable in the Dutch banking sector, where one of the market dominating banks, the Rabobank, had already installed an EC in 1998 ([26, 33]; www.rabobank.com). Other banks, such as ABN Amro and De Volksbank, followed this example in the preceding decade (www.abnamro.com; www.devolksbank.nl). To offer a reflection platform for all banks about overarching ethical issues, an inter‐banking ethics group was also established in the Netherlands (www.nvb.nl). In the German banking sector so far, only the Deutsche Bank makes use of a comparable structure, the so‐called “Integrity Committee of the Supervisory Board of Deutsche Bank Aktiengesellschaft” (www.db.com). Up to now, literature and scientific findings about the nature of ECs in the banking sector were very scarce.

From 2011 on, ECs in the scientific research sector that focused on dual use research of concern were about biosecurity in the life sciences. One specific target was a response to the work of two research groups on the transmissibility of the H5N1 virus, commonly known as avian flu [19], one at the University of Wisconsin in the US and another at the Erasmus Medical Center in the Netherlands. Given the potential misuse of the avian flu virus, which had a mortality rate of 60%, the US National Science Advisory Board for Biosecurity was asked to provide guidance for publications about this research area [19]. In 2012 the World Health Organization announced that these publications might benefit the creation of mechanisms to deal with other dual use research information in the future [18].

In response to these developments in the European setting, specifically in Germany, the German Ethics Council studied the subject of biosecurity freedom and responsibility and came up in 2014 with an opinion paper on biosecurity (www.ethikrat.org). In addition, from 2014 on, the German Research Foundation (DFG) and the German National Academy of Sciences Leopoldina established a joint committee that works out recommendations for dealing with security relevant research and also helps to implement these recommendations at German universities and research institutions (www.leopoldina.org). Since these ECs focusing on dual use research of concern are currently evolving, scientific findings and literature about their nature is still very scarce.

## 3 Methods

### 3.1 Research Design

Due to the explorative nature of the main research question used in this study, our research approach used here represents a constructivist view, which emphasizes that social and psychological worlds are constructed, and sense‐making is created through social processes and interaction (e.g., [34, 35, 36]). To understand which EC practices were identified by the participating EC experts as being of particular relevance for the installation of well‐established ECs, we used the following inductive approach for data gathering. In a first phase we conducted focus group expert interviews to gather an overview on a more general level about the EC practices mentioned. In a second phase, we held individual, in‐depth interviews among the EC experts who participated within the focus group interviews. These interviews gathered more detailed information about why in the first phase, EC practices were identified as having particular relevance for the installation of ECs.

### 3.2 Study Setting

We examined our research question using EC experts who came from the healthcare setting, from the banking and from the scientific research setting. These EC experts formed part of an EC operating on a non‐mandatory basis that gave advice to all organizational stakeholders who submitted an ethical question from clinical work practice, from the banking work practice or from the scientific research work practice.

### 3.3 Recruitment and Design

For the identification and selection of information‐rich cases (e.g., [37]; Sekaran, Bougie [38]), we used a purposeful sample that was recruited by an email invitation to participate in this explorative study about practices of ECs in different sectors.

The candidates from the healthcare sector were contacted based on a publicly available list of clinical ECs published by the German Academy of Ethics in Medicine (AEM) in 2022. Candidates from the scientific research sector were also contacted by an e‐mail invitation based on a publicly available list of ECs focusing on dual use research of concern by the German National Academy of Sciences Leopoldina in 2022. Experts for ECs operating in the banking sector formed part of the authors' professional network in the Netherlands and were also contacted by an e‐mail containing an invitation to participate in this study. The criteria for selection were the following:

- All experts are or have been members of an EC.
- Chosen clinical ECs have existed longer than 5 years and form part of a university hospital.
- Chosen ECs operating in the banking sector have existed longer than 5 years except for one expert who operates in a newly established EC but has extensive knowledge about ethics management in banking.

#### 3.3.1 Focus Group Interviews

The focus group interviews were organized consisting of in total (*N* = 4) experts from banking ECs, (*N* = 2) experts from clinical ECs, (*N* = 2) experts from the scientific research setting in three discussion rounds. For an overview about the main characteristics of the participating EC experts see Table 1.^6^

**Table 1:** Main characteristics of participating ethics committee experts.

Expert	Sector	Country	Size of the organization	EC membership
Expert B01	Banking	Netherlands	Multinational, large	Yes
Expert B02	Banking	Netherlands	Multinational, large	Yes
Expert B03	Banking	Netherlands	Multinational, large	Yes
Expert B04	Banking	Netherlands	National, large	Yes, for upcoming ethics committee
Expert R05	Research	Germany	National, large	No, but co‐worked with various national ethics committees
Expert RH06	Research, Healthcare	Germany	National, large	Yes
Expert H07	Healthcare	Germany	National, large	Yes

Each discussion round consisted of three phases. In the first phase, all participants were introduced to an ethical challenge case coming from either the clinical, banking, or scientific research setting. In the second phase, only the experts in the field of the ethical challenge case that was discussed got into dialog about how they approach such an ethically challenging case in their work practice. In the third phase, all participating experts were invited to contribute to the discussion. In these discussion rounds or focus group interviews, the discussion included practices, similarities, and differences among ECs across the healthcare, banking, and scientific research sectors. The discussion started with the main question of which practices can be observed in general and then followed with discussion rounds that concentrated on the differences or similarities and on practices for ECs. For an overview of the structure and questions used for the investigation during the focus group interviews as well as the used ethically challenging cases please see the Appendices A, A1, A2 and A3.

#### 3.3.2 Semi‐Structured Individual Interviews

After the three focus group interviews, the first author conducted six semi‐structured individual interviews with experts who participated in the focus group interviews^7^. These individual, in‐depth interviews complemented and produced further insight into the data gathered from the focus group interviews. The individual interviews focused on the main dimensions inductively derived from the data of the focus group interviews. For an overview about the interview guidelines and questions used at the individual interviews see Appendix B.

### 3.4 Analyses

The focus group interviews and individual interviews were transcribed *verbatim*. The data was analyzed thematically on content in line with the quality standards in the literature [39]. This means in an initial phase, the main thematic categories derived from the main research question as mentioned above were used to code meaningful fragments in the data while reading it closely. Then all fragments belonging to the same main category were compiled to create sub‐categories derived inductively based on the data. This phase was followed by a second coding phase in which all the data was coded using the elaborate category system. In this phase of the analysis, we focused in particular on the dynamics or, in other words, the positive or negative reactions that the experts participating in the roundtable discussions seemed to experience or showed as a result of the discussions among these experts.

To structure our analysis, we used the analysis program MAXQDA. To increase reliability, all transcripts and reports were coded and analyzed by at least two researchers. We also used respondent validation by discussing the themes and categories found in the focus group interviews with the experts in the in‐depth interviews. Analyses, themes, and categories were repeatedly discussed by the research team until consensus was reached.

### 3.5 Ethical Considerations

Before the start of the study, the study concept received an official positive review by the research ethics committee at the *Charité‐Universitätsmedizin* Berlin. All participants were informed both orally and in written form about study procedures, confidentiality, and privacy procedures. All participants signed an informed consent form and gave permission for the interviews to be recorded and for the data from the interview to be published. The research data were stored in a secured project file. Access to study data was limited to members of the research team. Audio and video recordings were destroyed after transcripts were made.

## 4 Results

As an overarching statement, it can be said that it was not self‐evident that a dialog between EC experts coming from three different sectors can be fruitful. However, the results reveal that despite the differences between the sectors, the similarities prevail. The participants focused on a meta level for discussion, where the focus is more on how differences in EC practices can be transferred from one sector to another and what are the similarities between the participating sectors, for example as reported by expert B01 by saying:

“… We've been talking about this kind of mini conference a lot, … but sometimes I was a bit scared, [if] … everyone is willing … to look further than their own discipline or their own context or sector? But that was very stupid of me because from the start, I think everyone naturally focused on the overarching things and, … that is hopeful in the sense that … even though there might be some… barriers, there is no barrier to talk about ethics …”

In sum, we were able to distil two main thematic categories – *moral authority* and *trustworthiness*, and three sub‐categories – *legitimation*, *mode*, and *outreach* on a meta level for the ECs in which the participating experts described in the following paragraphs operate.

### 4.1 The Main Thematic Categories: Moral Authority and Trustworthiness for ECs

Issues of moral authority, which represents “the ability or power to make someone act ethically”, and moral trustworthiness of an EC's advice, which can be described as “the ability to convince someone of the normative robustness of an EC's given advice”, are the main concerns raised by the expert panel.

Establishing ethics in organizations in an institutionalized form is not a given in the clinical, the research, or the banking sector. This can have different reasons depending on the sector. For example, ethics in the clinical and research context might be understood as being situated foremost in a professional ethos and as an individual responsibility. In this vein, the respective codes of conduct are aimed at individual behavior by clinicians and researchers and are by nature embedded into the organizational structure. In the banking sector, the focus on shareholders and other stakeholders interests might represent an obstacle to understand ethics as a natural structural element of an organization. In this vein, for example, transactional shareholders, who seek to maximize short‐term financial returns with little regard for nonfinancial goals, can have a significant influence on the interests of other stakeholders and ethics in the organization. For example, several studies show that transactional ownership can disadvantage employees by reducing employment security and job prospects (e.g., [40]). Noteworthy, also in the scientific research sector, there may be instances where ethical concerns and the security measures that are ethically justified could potentially conflict with the freedom of research (e.g., [41]).

Therefore, it is not self‐evident that an EC would be established within the organization, that its services are requested and that any advice it gives is also perceived, implemented, and viewed as a morally important part of an organization culture. The conditions for establishing and maintaining well‐functioning ECs thus lies in the intersection of moral trustworthiness on the one hand, and moral authority on the other. To safeguard both aspects, organizations have their own challenges as will be shown in the next paragraph.

### 4.2 The Subcategory EC's Added Value or Legitimation

As mentioned, the participants in the expert panel expressed their concerns about how an EC can legitimize itself within an organization. The prevalence with which this requirement is discussed in the focus group and individual interviews shows that it is often not or at least not completely clear to every organizational stakeholder which role or function the EC plays in the organization, although it is visibly installed in this organization. It seems that when the board of directors gives an EC the mandate in the organization to be concerned with ethical challenges, such a step is necessary but insufficient to promote the EC's legitimacy.

A common reaction that arises here is an ongoing discussion about the EC's added value or the EC's legitimation within the organization. Such a reaction would be especially likely for those ECs in the scientific research setting that are concerned with dual use questions, which represent a rather new form of ECs within the research sector compared to other pre‐existing ECs such as IRBs. The main challenge here lies in increasing the visibility or more precisely in raising the awareness that there might be an ethical issue within an individual research project. Hereby, the role of awareness raising was described as intertwined with the provision of training, as highlighted by, among others, expert R05, for example in the following phrase:

“… one of the most important aspects … is the training and awareness raising of the scientists themselves about the work that they might carry out.”

Furthermore, all participants agreed that a necessary condition for an EC legitimation by all its stakeholders is that advice requesting must fit into other standard operating procedures of the organization, for example the review procedures used by ECs should not lead to overly complicated bureaucratic efforts for the requester. In this vein it was put forward by an expert coming from the healthcare and research setting (expert RH 06):

“… very important is … [the] reviewing procedure, if it is going bureaucratic, they will not come to us, because they have no time [and do not see the added value] …”

It was also reported by expert RH 06 that another factor was crucial to enhance the visibility of the EC's added value to the organization. That factor was to be in continuous dialog between EC members and advice requester(s) to achieve a shared understanding for the legitimacy of the EC. A phrase that highlights these is the following:

“… there is really some sort of mistrust … always a good thing is, if you are working together on a … interdisciplinary project … and that the other[s] … gets to know what you are dealing with … then it gets easier … then one person comes to you as an ethicist and says: “… I need your advice” … this is a special way of having to do with each other …”

This response also reveals that the continuous dialogs that are regarded to be key to the EC legitimation process will pursue the goal of building a relationship between the advice‐requester and advice‐giver. This relationship becomes one where trust in the integrity of the EC member giving the advice plays a substantial role.

The account given above indicates that the establishment of an EC's moral authority and moral trustworthiness lies in achieving three goals:

1. Offering adequate awareness and training opportunities to all EC stakeholders such as managers, employees, and customers, among others.
2. Showing that the EC procedures fit well into the organizational standard procedures.
3. Demonstrating its capacity to build up and maintain an ongoing dialog and relationship between the advice‐givers and the advice‐requesters based on trust, especially trust in the integrity of the EC members.

### 4.3 The Subcategory Mode: Reactive or Preventive

Another challenge for an EC that the participants of the expert panel discussed has to do with the dynamic aspects of the emergence, development and ultimately the handling of an ethical question or challenge. In comparison to other areas in an organization, such as standard operating procedures in quality management, an ethical challenge that arises in an organization can take different forms:

- The standard form is that an ethical challenge exists and is recognized as such by the members of an organization. The EC is here requested in a reactive way for advice.
- The ethical challenge may exist without being recognized by the members of the organization, which makes a screening method necessary.
- The ethical challenge may exist, but is only recognized by certain stakeholders or only by stakeholders outside the organization. In this context, an EC can serve as a moderator, overseeing the continuous moderation of ethical challenges between different stakeholders.
- The ethical challenge has not yet arisen, but it has already been recognized that a specific situation could become ethically relevant which represents the preventive mode.

A reactive mode implies that the EC exclusively reacts to incoming ethical challenges often in one single point in time. A preventive mode implies that the EC looks for ethical challenges before they arise. As for example reported by expert B02 by the following citation:

“… there is maybe a little difference of opinion within … the ethics committee that I experienced when … to create kind of a model for forward looking, … that some people in the committee really like to discuss the more concrete … individual cases [in retrospection looking back] and others … they also want to really look into the future [in prospection looking forward] … and then of course, environmental or social issues that have a high urgency, such as climate change …”

In a reactive as well as preventive mode can be realized by operating as a moderator for ethical challenges as for example reported by expert B04 from the banking sector:

“… in an agile organization, in which we work … we also have with the data or with some kind of AI application …possible negative consequences. So … we start with minimal viable products and then work on a stop or go. So, we, every week … discuss, “Okay, does this product really, you know, serve our purpose in what we want? Do we serve the society as much as we can? Is the … impact on society, the negative impact on society minimal? And how can we safeguard that minimal negative impact? …”

All participating EC experts reported for the ECs in which they operate that they give advice within existing cases in what can be called a reactive mode. They also reported about EC actions by screening ethical challenges and addressing potential ethical challenges within the frame of ethics trainings, EC actions that can be regarded as representing the preventive mode. Besides this, based on the results of this study, it can be stated that in the clinical ECs there is still more a tendency to discuss existing cases, which means using the reactive ethical challenge handling mode for at least part of the time. The banking ECs also regularly reported discussing upcoming ethical trends or risks resulting in a preventive EC mode. However, in the scientific research setting the discussions are continuously focused on potential ethical risks resulting in a preventive EC mode, exclusively.

Bearing in mind the results above, the first impression that comes to mind might be that an advice‐giving approach in the EC that aims towards a preventive mode for an EC's actions is preferable since it might increase the chances of preventing of some or even many ethical challenges. Universally applying a preventive mode can also be regarded as short‐sighted, since a reactive mode for EC action also has specific uses in certain cases or situations. For example, as mentioned above the emergence of an ethical challenge might not be always visible immediately and could evolve or change over time and by location. In addition, there are also currently existing ethical challenges in organizational practice that require a reactive mode for EC action or advice‐giving approach to overcome and to lead to a preventive mode for potential future ethical challenges.

Given the above account it rather seems that an approach that takes various possible modes (reactive, screening, moderating, and preventive) into account can have a positive effect on decision‐making quality in an EC.

It was noteworthy that the experts from all three sectors reported that they make use of a casuistic approach or situation‐specific analysis to come up with advice for ethically challenging cases. This casuistic approach or situation‐specific analysis approach is described by the experts from the healthcare and scientific research setting as an approach based on principle ethics while the experts from the Dutch banking sector talked here about the establishment of “moresprudence”. The meaning of moresprudence is that ethical knowledge gathered by advice given to prior similar ethically challenging cases like the current one at hand do influence the newly given advice based on practical wisdom. This approach to ethical advice giving also provides some indication that a sequential approach to ethical advice giving occurred in practice on a long‐term basis, although not at the level of following up the given advice to the one specific ethically challenging case at hand.

Putting these in light of the main thematic categories found for requirements when establishing and maintaining ECs, it can be assumed that it is possible with every given advice by the EC that the here so‐called and described situations‐specific approach leads to stronger foundations for the advice given by the EC. These stronger foundations can be one of two types: an enhancement of both moral authority and trust given to the EC by different stakeholders, or the enhancement of such moral authority and trust given by at least the advice‐requesters.

### 4.4 The Subcategory Outreach: On a Micro, Meso, and/or Macro Level

Based on the comparative analyses we also found that the outreach of an ethically challenging case can know different levels. The levels that can be distinguished here are the micro level in which an individual advice‐requester, for example a patient, client or researcher asks for ethical advice, the meso level where ethical advice is given that affects for the most part the organization at hand internally, and a much broader macro level in which the given advice by the EC can affect a very broad audience on a societal level as for example pointed out by expert B04:

“… it should evolve from a discussion in the interbank ethics group. So, if in the ethics committee, we often flag an issue saying, we can only solve this at the interbank level. Then we bring it to the inter banking discussion. And if … we derive in a position like this would be good for society then, I think it is our responsibility to bring it to our boards, respective boards and make it happen …”

This finding gives implications that the engagement of all relevant stakeholders can be crucial to ethical challenge resolution. For example, as the citation above also illustrates, some ethical challenges might occur or become visible at a micro/individual case level but ask for ethical advice on an organizational or institutional or even broader societal level for resolution. One promising approach can be that representatives of all relevant stakeholders such as patient, client, researcher representatives, but also representatives of different organizational departments such as finance and human resources make up part of the EC, leading to a very diverse EC composition for its members. In addition, the results of the study give some indications that interorganizational institutional structures at national or even international level are required for the resolution of some ethically challenging cases.

The results above reveal that to safeguard ethical challenge resolution and by this ECs' moral authority and trustworthiness, it is necessary that they do not operate in organizations as self‐contained entities, but that they rather reach out and communicate with all relevant stakeholders on micro, meso and macro levels about previous, contemporary, and upcoming ethically challenging cases.

## 5 Discussion

In this study, we were guided by the main question: *“Based on a comparative, cross‐sectoral approach to investigate ethics committees, what are the main requirements for the functionality of ethics committees?”*. As such, this study represents more than the first contribution to the scientific literature of ECs that compares practices in different sectors. This study is also the first contribution that identifies *moral authority* and *trustworthiness* as two broad main requirements and the three underlying categories: *legitimation*, *mode*, and *outreach* to establish ECs that can run according to their anticipated function in different sectors.

For each of them we have described the results and discussed how they might affect ECs' advice‐giving character for clinical, banking, and research practice. Hereby, it needs to be noted that since ECs emerge in different settings and different forms, most choices made with respect to the found requirements are very context‐specific. It is therefore difficult to describe specific recommendations that match with the needs of every EC: there is no one‐size‐fits‐all solution.

Nevertheless, based on the comparative analyses as described above we could distil the following requirements for ECs' legitimation, mode, and outreach:

Requirement 1 *Building and maintaining the dialog by awareness as well as training campaigns, fitting into organizational standard procedures, and creating trust in the integrity of the EC members all have a positive effect on ECs'perceived legitimation by relevant stakeholders*.

There already exists an extensive amount of scientific literature which puts forward offering awareness and training opportunities to different kinds of stakeholders as an EC main function (e.g., [1, 24, 28, 42]). In practice it seems that the bigger challenge with the traditional awareness campaigns and training opportunities (e.g., [43]) is not to offer such campaigns and opportunities but rather their restricted uptake leading to restricted effects.

Another striking finding of this study is that EC procedures need to fit well into other organizational standard procedures because otherwise the setup can lead to low submission rates of ethically challenging cases. This can lead to a lower uptake of ethically challenging cases in the EC, even though they exist in the organization. While this finding might sound simple, and also, we already know from previous research findings that resistance to submit ethically challenging cases to the EC can affect EC success (e.g., [44]) according to the participating EC experts, in practice this relatively huge challenge still remains.

Therefore, this finding can be assumed to highlight one more time the importance that ECs in organizations do not perceive themselves and are not perceived by other organizational stakeholders as self‐contained entities where somehow all ethics related topics can be left behind. Rather, ECs need to be in continuous communicational exchange or dialog with the end user, different departments in the organization, and external stakeholders (e.g., [16, 45]). ECs also need to adapt their structures according to the existing needs. Hereby, ECs should be seen as being only one component of a whole ethics program: they represent an additional organizational layer to promote ethics in the organization. ECs based on a non‐mandatory basis do support, complement, or replace existing organizational structures. Therefore, ECs need to be regarded as an additional safety mechanism in the organization.

Moreover, when it comes to creating trust in the integrity of the EC members to enhance EC legitimation, there already exists an extensive amount of scientific literature that show that member expertise (e.g., [46, 47, 48]) and member diversity are crucial factors (e.g., [46, 49, 50, 51]) for ECs legitimation and its success. A precondition to realize this ideal member expertise and diversity composition within an EC was already found by Beyleveld, Brownsword and Wallace [52] that an EC should not only be aware of its declared mission, but also about how to put it into practice. In other words, it is of importance that the EC understands its role within the organization. Otherwise, it might be difficult to fulfill the anticipated EC function within the organization and maintain trust in the EC. Thus, having an EC is not sufficient to safeguard ethics in the organization. Its value is highly related to its composition and the ability of its members to promote the EC's mission. Therefore, finding the right match between EC mission and function with its members is crucial to its survival.

Requirement 2 *An approach that involves various possible EC modes (reactive, screening, moderating, preventive) has a positive effect on the quality of the EC's decision‐making process*.

The quality of the decision‐making process can be assumed to be enhanced by making use of an approach to ethical decision‐making that takes into account various possible modes (reactive, screening, moderating, preventive) since this leads automatically to the uptake of more ethically challenging cases. This increase occurs because the EC handles incoming ethical questions as well as screens and/or moderates for already existing or not existing yet but upcoming and/or potential ethical questions. This can be assumed to lead to rebound effects because an increased number of ethical questions leads to an increased decision‐making quality by the above‐described situation‐specific approach to ethical decision‐making.

Requirement 3 *Reaching out to all relevant stakeholders leads to enhanced ethical challenge resolution capacities*.

Outreach can be implemented at different levels depending on the estimated necessity for relevant stakeholder engagement. Such multi‐level implementation can create a more transparent information sharing process among all stakeholders such as managers, employees, and customers, among others. This in turn can be assumed to lead to a higher chance for the establishment of a “speak up” or “open failure culture” which leads to an increased submission of ethically challenging cases and trust given to EC advice (e.g., [53, 54, 55]).

### 5.1 Strengths

The systematic, iterative way of collecting and analyzing the data as well as making use of respondent validation and peer review in this study in line with Hennink [56] and Kuckartz [39] helped us to verify the results and interpret them in various complementary rounds. In addition, this study is the first contribution that takes into account and compares ECs' practices in various sectors. Nevertheless, this study is more intended to represent a first explorative, general guide for potential main requirements when establishing and maintaining ECs in different sectoral and organizational settings. This approach comes with some limitations.

### 5.2 Limitations and Implications for Future Research

First, although we tried to select information‐rich expert cases by the used sample selection strategy as described above, the sample size was rather small and not chosen randomly. This makes the assessment of the generalizability of the study questionable (e.g., [57, 58, 59]). It should be noted, however, that in a qualitative and exploratory study following a constructivist paradigm, generalizability in a statistical sense is not an aim per se and not the crucial point when assessing the quality or validity of a qualitative study [39]. Through the chosen design of the study (data collection in several rounds based on focus group as well as individual in‐depth interviews) and the method of analysis through the use of peer review as well as respondent validation, we still tried to increase the transferability or generalizability of the study results.

This study also posed questions that we did not have enough resources to answer. More research is needed. For example, we did not address the topic of monitoring ECs and the requirements for specific expertise and qualifications needed by EC members.

In this study we also focused exclusively on ECs in the healthcare, banking, and scientific research sectors that serve the function of advice giving to clinical, business, and research practice respectively. But because as described above, ECs emerged in various sectors (e.g., [16]), it might be possible that ECs in other sectors and organizational settings serve different functions.

Future research needs to focus on the further validation of the found study results by using empirical research based on a large scale and longitudinal design. Besides this, this study was conducted in the German healthcare, German research, and Dutch banking sectors. Some results might have been affected by the national context, therefore it is recommended that the found requirements are further validated in a non‐European context, for example, the US or a country in Asia.

### 5.3 Potential Implications for Practice

Overall, the results of this study reveal that ethics in organizations is not self‐evident. Specifically, the installation as well as uptake of ECs operate in a field of tension. This field of tension moves between dimensions that are concerned with how powerful an EC is in making someone act ethically and how trustworthy an EC is in convincing relevant stakeholders on the normative robustness of EC's advice given. In addition, the requirements that are found concerning EC legitimation, mode, and outreach can be used as areas of concern by organizations in different sectors during the process of installing an EC with an advice‐giving character.

## Conflicts of Interest

The authors declare no conflicts of interest.

## Data Availability

The data that support the findings of this study are available from the corresponding author upon reasonable request.

## Endnotes

1 To be defined in this study according to Kaptein and Schwartz (2008: 113) as “a set [of formal] prescriptions developed by a company [or organization in general] to guide the behaviour of managers and employees.”

2 An anonymous reporting tool that allows employees to report and potentially expose illegal, unethical, and/or improper conduct.

3 Based on a questionnaire that was non‐sponsored and unsolicited, sent to the top 500 companies operating in the private sector within Australia (Business Review Weekly 2005), Canada (Financial Post 500 2005) and the United States (Fortune 2007).

4 The forerunner of contemporary bioethics committees evolved in 1962 in Seattle and was called the “Life or Death Committee”.

5 A court case in the US in which the parents of a woman who was kept alive by artificial means were allowed to order her removal from artificial ventilation.

6 It needs to be noted that one participating expert (expert RH 06) represents in this study a double role as expert from both the healthcare and scientific research sectors.

7 Due to illness, it was not possible to conduct the individual, in‐depth interview with one of the experts (expert H07) coming from the healthcare setting.

13 * Based on Varkey [25].

14 * Based on Rost van Tonningen and Louwers [26].

15 * Based on Himmel [21].

## Appendix A Structure of Focus Group Interviews

**Table:**

09.00 am – 10.45 am	Introduction by the moderator of the day.
10.45 am – 11.00 am	*Short break*.
11.00 am – 11.45 am	*Discussion round 1*: Case number one – ethically challenging case in the scientific research setting (all participants receive a hand‐out with the case description) *Main question*: “Which practices for dealing with ethical questions in organizations, supported by ethics committees, can be formulated across the healthcare, banking and scientific research sectors?” *1.1 Discussion in the clinical group about the following question*: “Which practices for dealing with ethical questions in organizations, supported by ethics committees, can be formulated?” → the others observe and make notes. *1.2 Discussion about the results round two in the whole group concerning the following sub‐questions*: *Sub‐question 1*: “Which similarities and differences concerning practices for ethics committees between the healthcare, banking and scientific research sectors can be identified?” *Sub‐question 2*: “Which main practices can be formulated for ethics committees in the healthcare sector (clinical setting)?”
11.45 am – 12.30 pm	*Discussion round 2*: Case number two – ethically challenging case in the clinical setting (all participants receive a hand‐out with the case description) *Main question*: “Which practices for dealing with ethical questions in organizations, supported by ethics committees, can be formulated across the healthcare, banking and scientific research sectors?” *1.1 Discussion in the banking group about the following question*: “Which practices for dealing with ethical questions in organizations, supported by ethics committees, can be formulated?” → the others observe and make notes. *1.2 Discussion about the results round three in the whole group concerning the following sub‐questions*: *Sub‐question 1*: “Which similarities and differences concerning practices for ethics committees between the healthcare, banking and scientific research sectors can be identified?” *Sub‐question 2*: “Which main practices can be formulated for ethics committees in the banking sector (banking setting)?”
12.30 pm – 13.15 pm	*Lunch break*
13.00 pm – 13.15 pm	*Meeting moderator, co‐moderators, observers and note takers*
13.15 pm – 14.00 pm	*Discussion round 3*: Case number three – ethically challenging case in the banking setting (all participants receive a hand‐out with the ethical case description). *Main question*: “Which practices for dealing with ethical questions in organizations, supported by ethics committees, can be formulated across the healthcare, banking and scientific research sectors?” *1.1 Discussion in the banking group about the following question*: “Which practices for dealing with ethical questions in organizations, supported by ethics committees, can be formulated?” → the others observe and make notes. *Sub‐question 1*: “Which similarities and differences concerning practices for ethics committees between the healthcare, banking and scientific research sectors can be identified?” *Sub‐question 2*: “Which main practices can be formulated for ethics committees in the banking sector (banking setting)?”
14.45 pm – 15.15 pm	*Short break*
15.00 pm – 15.15 pm	*Meeting moderator, co‐moderators, observers and note takers*
15.15 pm – 16.30 pm	*Lessons learned and rollout*: *Questions here*: “What have been the lessons learned/eye openers about practices for ethics committees from the banking, healthcare scientific research perspectives?” “Are there any questions left we have forgotten to ask or a topic concerning practices that have not received sufficient attention?” (last notes from every participant)

## Appendix A1 Ethically Challenging Case Example From the Scientific Research Setting

Ethics in scientific disciplines have generally been characterized by several core principles including: “Honesty in reporting of scientific data; …analysis of scientific results to avoid error; …interpretation of results that is based on data and not on the influence of external sources; Open sharing of methods, data, and interpretations…; …validation of results through replication and collaboration with peers; proper crediting of sources of information, data, and ideas; Moral obligations to society in general…”^8^

Over the past two decades, the results of various studies undertaken in the life sciences and related fields that have been published in scientific journals have increasingly caused security concerns that are centered around the issue of *dual use*, that is, work that provides results that are not only beneficial, but that can be misused to cause harm. As a result of this work ethical considerations in the life sciences have been extended beyond the usual core principles to include ethics based on security‐relevant issues.

Some of this work has caused *particular* concern and is known as “dual use research of concern” or DURC. DURC is generally defined as “Work in the life sciences that can provide knowledge, products or technologies that can be misused to cause extensive harm to humans, plants, animals or the environment.”^9^ An example of such work can be used to describe a dual use dilemma in science:

The avian influenza virus designated H5N1 causes a severe disease with high mortality (60% of the infected) in birds (fowl), but is not easily transmitted to humans, and when, then usually through direct, physical contact with infected birds, resulting in a similarly high mortality. In 2012, a group of researchers in the Netherlands^10^ and another working in Japan and the USA^11^ experimented with the highly pathogenic H5N1 virus, producing mutated (genetically changed) variants of the virus that could infect mammals (in this case ferrets) through airborne transfer. The researchers had no malign intent by carrying out this work. Their stated aim was to determine what mutations could alter the host range of the virus and enable it to be transmitted through the air from individual to individual; this, to be better prepared for such changes that might occur in nature. Nevertheless, they had in effect created a potentially highly dangerous virus for humans, although the effect on humans could of course not be directly tested.

This work provoked a heated debate in public and scientific circles as to whether it should have been carried out at all, both for safety (the virus could escape the lab) and security reasons (a blueprint for those with malign intent for creating viruses with similarly dangerous characteristics).

In general, this type of work has been designated gain of function (GOF) research on highly pathogenic microorganisms, as the virus in this case gained characteristics it did not have before: an alteration in host rage (from fowl to mammals) and the ability to be transmissible.

## Appendix A2 Ethically Challenging Case Example From the Clinical Setting

*From the mind of a salesperson* 12. A 25‐year‐old woman has been chronically ill since birth with mucoviscidosis (cystic fibrosis), which causes thick mucus and other fluids to build up and clog different parts of the body, including the lungs and digestive tract. She is very physically active and lives with her parents, by whom she is lovingly cared for. Many medical treatments and several hospital stays since childhood have made the young woman fearful of doctors and hospitals.

Several weeks ago, her family doctor diagnosed her as having a hernia. He recommends an operation, but the patient refuses to go to hospital because she is afraid of the anaesthetic. The parents do not want to burden her unnecessarily. Her family doctor considers this acceptable but advises regular check‐ups because he sees a high risk of severe *paraesophageal hernia* in view of her physical activities.

While she is taking a shower, the patient discovers a lump in her breast. After examination by her doctor, he urgently suggests surgical removal because a malignancy cannot be ruled out. The patient, who is fully legally competent, is then persuaded by her parents and is admitted to hospital for breast surgery. At the clinic, after speaking with the patient about it, the parents express the wish to operate on the inguinal hernia when she is under the same anaesthetic. From a medical point of view, the risk of anaesthesia is increased with mucoviscidosis. The gynaecologists agree and call in the general surgeons. The surgeons, then refuse the simultaneous operation. Since it is not yet clear whether the tumor is benign or malignant, the bills for the operations are to be compared based on the relevant base case value:

1. Mammary segment resection for malignant neoplasm (a procedure to remove cancer from the breast, but not the breast itself, and any regular tissue around it) with or without rupture surgery as a secondary diagnosis: revenue 4019 €.
2. Mammary segment resection for benign neoplasm (a procedure to remove a benign tumour from the breast, but not the breast itself, and any regular tissue around it) with or without hernia surgery as secondary diagnosis: revenue 2065 €.
3. Inguinal hernia operation alone (surgery to repair a hernia in the groin): revenue 2181 €.

The hernia operation in combination with the mammary segment resection: does not affect revenue.

In the case of two separate operations, the hospital would thus earn € 2181 more, but would of course also have somewhat more expenses. Case splitting is not feasible even with a short time interval between the operations, as the two conditions are independent. Case splitting is therefore financially worthwhile and would contribute to more revenue for the department. The parents and the patient are told that it does not make sense to perform both operations at the same time because they differ in the risk of infection (“clean” breast operation and potentially “unclean” hernia operation). The urgent breast operation should be performed first, and the inguinal hernia operated on after the breast has healed. The young woman and her parents agree to this procedure.

Since the paediatrician and adolescent doctors are also looking after the patient because of her chronic illness, the case comes up a few days later at a meeting between them and the hospital's financial director. The director then says: “You can't do that!”

## Appendix A3 Ethically Challenging Case Example From the Banking Setting

*The rental mortgage*. With the current surplus of liquid assets (savings) and the desire to promote the bank's profitability, the bank “*new generation*” is looking for additional ways of placing money with a good commercial margin at the market.

At the intersection of the private and the business spheres, a new opportunity has been found for lending money. That new opportunity is to lend, on a mortgage basis, to private individuals who wish to purchase properties with the aim of renting them out to third parties (instead of living there themselves or keeping them as holiday homes, etc.).

The current increase in wealth was generated by several factors, including cashing in of excess value on one's own home and/or saving money. Both factors were in part fueled by the Corona pandemic. The private individuals who owned part of this excess wealth faced a low return on savings and bonds, and thus needed to diversify their investments through real estate.

The aim of this product is to enable private individuals to buy a house for the purpose of renting it out. Thus, the main ethical risks identified for this product focus on the question of whether the introduction of this product would reduce the size of the current market value of houses in the Netherlands. Even if the market's size were to be reduced only in current public opinion, it still could have a negative effect. The bigger question is to what extent this goes against the wishes of society.

The action options offered for this case make part of the following broader ethical frameworks:

- The product could have the consequence of reducing the total number of available dwellings in the Dutch market (i.e., further increasing the existing absolute housing shortage).
- The product could have the direct effect of driving up the rental price of the property (i.e., increasing the “financial shortage” in the market).
- The bank “new generation” would assume a certain degree of responsibility towards the tenants about the quality and safety of the premises that they finance for rent, and possibly also responsibility about sustainability of the house (e.g. depreciation or deterioration).

## Appendix B Interview Guidelines and Questions for the Individual, Semi‐Strcutured, In‐depth Interviews Among EC Experts

**Part 1: Introduction**

All interview partners receive beforehand an overview about the main findings from the focus group interviews by means of a mind map created as notes during the focus group interviews.

The first author says some words of appreciation for the participation at this individual interviews. The first author explains briefly the three different types of ECs: the research, clinical and those existing in the banking sector, that participated in this study.

The interview partner is asked one more time for the permission for a voice record of the upcoming interview and receive a brief introduction into the anonymous process of data analyses in this study.

**Part 2: Middle part**

- Topic 1: Policy
  1. Do you make use of a code of ethics during your decision‐making process within the EC?
  2. Do you distinguish a code of ethics from a code of conduct in your organization? How do you safeguard the translation of formal code of ethics into daily work practice?
  3. Are these guidelines updated regularly? If so, how?
  4. Did you see any differences in the healthcare, banking or scientific research sectors' policies compared to your approach?
- Topic 2: Moral Authority and outreach
  5. Which mandate does the EC in your organization have – or with which governance structure in the organization is it aligned with? Would you say the EC is an independent structure in the organization?
  6. Can you describe the mission and vision of the EC in your organization in two or three sentences? Would you say the EC is successful in achieving this mission and vision?
  7. Is the EC visible and easily accessible in your organization? How do you achieve this?
  8. Did you see any differences in the healthcare, banking or scientific research sectors' EC mandates compared with your approach?
- Topic 3: Trustworthiness
  9. Would you say that the EC is highly supported by the leaders in your organization? Why?
  10. Is there and if so, which kind of resistance in your organization exists to come up with an ethical question to the EC?
  11. Did you see any differences within the healthcare, banking, and scientific research sectors in the way that they handle support or resistance to leadership?
- Topic 4: Decision‐making process
  12. In which form do you offer ethical awareness campaigns and training opportunities to all stakeholders of the organization? How do you evaluate their impact?
  13. Do you make use of a pre‐screening about ethical questions before they are reviewed by the EC?
  14. Which strategies (tools, methods etc.) do you use during the decision‐making of EC meetings?
  15. Which kind of methods do you use to measure, if the EC in your organizations is doing a good job?
  16. Which differences and similarities did you see between the decision‐making process in the healthcare, banking, and scientific research sectors?
- Topic 5: Committee mode
  17. How do you believe a responsible ethical, organizational culture can be fostered by means of an EC?
  18. Do you see differences between the healthcare and the banking sectors concerning the prospective viewpoint to come up with advice about ethical questions?

**Part 3: End**

19. If I really want to understand the contribution an EC can bring to an organization, did I forget to ask any question which could be of relevance within this frame?
20. What is your impression of this interview?

## References

[1] L. Farber Post and J. Blustein , Handbook for Health Care Ethics Committees (Baltimore: John Hopkins University Press, 2021).

[2] C. Grady , “Institutional Review Boards,” Chest 148, no. 1 (2015): 1148–1155. 26042632 10.1378/chest.15-0706 PMC4631034

[3] V. Bigo , “On Silence, Creativity, and Ethics in Organization Studies,” Organization Studies 39 1 (2018): 121–133, 2081.

[4] G. Martin , S. Bushfield , S. Siebert , and B. Howieson , “Changing Logics in Healthcare and Their Effects on the Identity Motives and Identity Work of Doctors,” Organization Studies 42, no. 9 (2021): 1477–1499.

[5] C. Rhodes , “Democratic Business Ethics: Volkswagen's Emissions Scandal and the Disruption of Corporate Sovereignty,” Organization Studies 37, no. 10 (2016): 1501–1518.

[6] C. Rhodes , “The Ethics of Organizational Ethics,” Organization Studies 44, no. 3 (2023): 497–514.

[7] A. L. Wright , G. Irving , and K. Selvan Thevatas , “Professional Values and Managerialist Practices: Values Work by Nurses in the Emergency Department,” Organization Studies 42, no. 9 (2021): 1435–1456.

[8] J. Greenberg , “Who Stole the Money, and When? Individual and Situational Determinants of Employee Theft,” Organizational Behavior and Human Decision Processes 89, no. 1 (2002): 985–1003.

[9] M. Kaptein , “Ethics Programs and Ethical Culture: A Next Step in Unraveling Their Multi‐Faceted Relationship,” Journal of Business Ethics 89, no. 2 (2009): 261–281.

[10] M. Kaptein , “The Effectiveness of Ethics Programs: The Role of Scope, Composition, and Sequence,” Journal of Business Ethics 132, no. 2 (2015): 415–431.

[11] J. B. Singh , “Ethics Programs in Canada's Largest Corporations,” Business and Society Review 111, no. 2 (2006): 119–136.

[12] L. K. Treviño , N. A. den Nieuwenboer , G. E. Kreiner , and D. G. Bishop , “Legitimating the Legitimate: A Grounded Theory Study of Legitimacy Work Among Ethics and Compliance Officers,” Organizational Behavior and Human Decision Processes 123, no. 2 (2014): 186–205.

[13] M. Babri , B. Davidson , and S. Helin , “An Updated Inquiry Into the Study of Corporate Codes of Ethics: 2005–2016,” Journal of Business Ethics 168, no. 1 (2019): 1–38.

[14] J. D. Moreno , “Ethics Committees and Ethics Consultants,” in Companion to Bioethics, eds. H. Kuhse and P. Singer (Oxford: Blackwell, 1998), 475–484.

[15] J. A. Robertson , “Ethics Committees in Hospitals: Alternative Structures and Responsibilities,” Issues in Law & Medicine 7, no. 1 (1991): 83–91. 1856074

[16] M. Verweij , F. W. A. Brom , and A. Huibers , “Do's and Dont's for Ethics Committees: Practical Lessons Learned in the Netherlands,” HEC Forum 12, no. 4 (2000): 344–357. 11138254 10.1023/a:1008997605664

[17] M. Callaghan , G. Wood , J. M. Payan , J. Singh , and G. Svensson , “Code of Ethics Quality: An International Comparison of Corporate Staff Support and Regulation in Australia, Canada, and the United States,” Business Ethics: A European Review 21, no. 1 (2012): 15–30.

[18] World Health Organization , 2012, *Report on Technical Consultation on H5N1 Research Issues*, http://www.who.int/influenza/human_animal_interface/mtg_report_h5n1.pdf.

[19] M. Magdalena Guraiib , A. L. Ross , A. Frewer , et al., “Oversight of Dual‐Use Research: What Role for Ethics Committees?,” Health Security 22, no. 4 (2024): 281–293. 38838255 10.1089/hs.2023.0095

[20] D. Patrone , D. Resnik , and L. Chin , “Biosecurity and the Review and Publication of Dual‐Use Research of Concern,” Biosecurity and Bioterrorism: Biodefense Strategy, Practice, and Science 10, no. 3 (2012): 290–298. 22871221 10.1089/bsp.2012.0011 PMC3440065

[21] M. Himmel , Biosecurity Risk Assessment in the Life Sciences: Towards a Toolkit for Individual Practitioners (Solna: Stockholm International Peace Research Institute, 2023).

[22] D. E. Hoffmann , “Evaluating Ethics Committees: A View From the Outside,” Milbank Quarterly 71, no. 4 (1993): 677–701. 8246852

[23] R. M. Lindsay , L. M. Lindsay , and V. B. Irvine , “Instilling Ethical Behavior in Organizations: A Survey of Canadian Companies,” Journal of Business Ethics 15, no. 4 (1996): 393–407.

[24] T. Arik , M. Kaptein , and E. Karssing , “Ethics Committee (Including Research),” in Encyclopedia of Business and Professional Ethics, eds. D. Poff and A. Michalos (Berlin: Springer, 2018).

[25] B. Varkey , “Principles of Clinical Ethics and Their Application to Practice,” Medical Principles and Practice 30, no. 1 (2021): 17–28. 32498071 10.1159/000509119 PMC7923912

[26] F. Rost van Tonningen and St. Louwers , Rabobank Ethics Committee: About Reflection Platforms, Moral Cost and Moral Case Law (Rotterdam: Nederlands Compliance Instituut, 2019), Retrieved from The‐benefits‐of‐ethical‐reflection.pdf (rabobank.com).

[27] T. W. Drew and U. U. Mueller‐Doblies , “Dual Use Issues in Research–A Subject of Increasing Concern?,” Vaccine 35, no. 44 (2017): 5990–5994. 28835345 10.1016/j.vaccine.2017.07.109

[28] G. McGee , J. P. Spanogle , A. L. Caplan , D. Penny , and D. A. Asch , “Successes and Failures of Hospital Ethics Committees: A National Survey of Ethics Committee Chairs,” Cambridge Quarterly of Healthcare Ethics 11, no. 1 (2002): 87–93. 11868423 10.1017/s0963180102001147

[29] K. Woellert , Praxisfeld Klinische Ethik: Theorie, Konzepte, Umsetzung am Universitätsklinikum Hamburg‐Eppendorf (Berlin: Medizinisch Wissenschaftliche Verlagsgesellschaft, 2021).

[30] E. Fox , S. Myers , and R. A. Pearlman , “Ethics Consultation in United States Hospitals: A National Survey,” American Journal of Bioethics 7, no. 2 (2007): 13–25. 10.1080/15265160601109085 17366184

[31] N. Steinkamp and B. Gordijn , Ethik in Klinik und Pflegeeinrichtungen (Köln: Luchterhand, 2010).

[32] M. Schochow , A. May , D. Schnell , and F. Steger , “Wird Klinische Ethikberatung in Krankenhäusern in Deutschland Implementiert?,” DMW ‐ Deutsche Medizinische Wochenschrift 139, no. 43 (2014): 2178–2183. 10.1055/s-0034-1370307 25317647

[33] M. Canoy , De bank van goede bedoelingen: De ramen open bij de Rabobank (Amsterdam: Uitgeverij Prometheus, 2019).

[34] K. Charmaz , Constructing Grounded Theory: A Practical Guide Through Qualitative Analysis (Los Angeles: SAGE Publications, 2006).

[35] K. Charmaz , “With Constructivist Grounded Theory You Can't Hide: Social Justice Research and Critical Inquiry in the Public Sphere,” Qualitative Inquiry 26, no. 2 (2020): 165–176.

[36] K. J. Gergen , “Theory of the Self: Impase and Evolution,” in Advances in Experimental Social Psychology, ed. L. Berkowitz (New York: Academic Press, 1985).

[37] E. A. Kemper , S. Stringfield , and C. Teddlie , “Mixed Methods Sampling in Social Science Research,” in Handbook of Mixed Methods in the Social and Behavioural Research, eds. A. Tashakkori and C. Teddlie (Thousand Oaks: Sage, 2003), 273–296.

[38] U. Sekaran and R. Bougie , Research Methods for Business: A Skill Building Approach (Chichester: Wiley, 2017).

[39] U. Kuckartz , Qualitative Text Analysis: A Guide to Methods, Practice, and Using Software (London: Sage, 2014).

[40] M. R. Des Jardine , M. Zhang , and W. Shi , “How Shareholders Impact Stakeholder Interests: A Review and Map for Future Research,” Journal of Management 49, no. 1 (2023): 400–429.

[41] P. Sikes and H. Piper , “Ethical Research, Academic Freedom and the Role of Ethics Committees and Review Procedures in Educational Research,” International Journal of Research & Method in Education 33, no. 3 (2010): 205–213.

[42] C. McDaniel , “Assessing Physicians' Roles on Health Care Ethics Committees,” HEC Forum 22, no. 4 (2010): 275–286. 21125312 10.1007/s10730-010-9142-5

[43] R. Stewart , B. Wright , L. Smith , S. Roberts , and N. Russell , “Gendered Stereotypes and Norms: A Systematic Review of Interventions Designed to Shift Attitudes and Behaviour,” Heliyon 7, no. 4 (2021): e06660. 33912699 10.1016/j.heliyon.2021.e06660 PMC8066375

[44] R. Førde and R. Pedersen , “Clinical Ethics Committees in Norway: What Do They Do, and Does It Make a Difference?,” Cambridge Quarterly of Healthcare Ethics 20, no. 3 (2011): 389–395. 21676326 10.1017/S0963180111000077

[45] M. S. McCabe , “Reenergizing the Hospital Ethics Committee: An Opportunity for Nursing,” Journal of Hospice & Palliative Nursing 17, no. 6 (2015): 480–487.

[46] A. Borovečki , H. Ten Have , and S. Orešković , “Education of Ethics Committee Members: Experiences From Croatia,” Journal of Medical Ethics 32, no. 3 (2006): 138–142. 16507656 10.1136/jme.2005.011643 PMC2564465

[47] A. F. Cook , H. Hoas , and K. Guttmannova , “Bioethics Activities in Rural Hospitals,” Cambridge Quarterly of Healthcare Ethics 9, no. 2 (2000): 230–238. 10742865 10.1017/s0963180100902093

[48] D. Milmore , “Hospital Ethics Committees: A Survey in Upstate New York,” HEC Forum 18, no. 3 (2006): 222–244. 17650761 10.1007/s10730-006-9009-y

[49] A. Matar and H. Silverman , “Perspectives of Egyptian Research Ethics Committees Regarding Their Effective Functioning,” Journal of Empirical Research on Human Research Ethics 8, no. 1 (2013): 32–44. 23485669 10.1525/jer.2013.8.1.32 PMC3712831

[50] R. Pinnock and J. Crosthwaite , “The Auckland Hospital Ethics Committee: The First 7 Years,” New Zealand Medical Journal 117, no. 1205 (2004): 1–10. 15570335

[51] H. Sleem , S. S. El‐Kamary , and H. J. Silverman , “Identifying Structures, Processes, Resources and Needs of Research Ethics Committees in Egypt,” BMC Medical Ethics 11, no. 1 (2010): 12. 20584332 10.1186/1472-6939-11-12 PMC2908628

[52] D. Beyleveld , R. Brownsword , and S. Wallace , “Clinical Ethics Committees: Clinician Support or Crisis Management?,” HEC Forum 14 (2002): 13–25. 12001798 10.1023/a:1020965130205

[53] G. Owen and G. Currie , “Beyond the Crisis: Trust Repair in an Interorganizational Network,” Organization Studies 43, no. 8 (2022): 1273–1295.

[54] B. L. Sadler and K. Stewart , Leading in a Crisis: The Power of Transparency (London: Health Foundation, 2015).

[55] J. Wilde , “Building Cultures of Transparency and Openness,” Implementing Culture Change Within the NHS: Contributions From Occupational Psychology 13 (2014): 64–79.

[56] M. M. Hennink , Focus Group Discussions (Oxford: Oxford University Press, 2014).

[57] G. Guest , K. MacQueen , and E. Namey , “Validity and Reliability in Qualitative Research and Data Analysis,” Applied Thematic Analysis (Thousand Oaks: Sage, 2012), 79–106.

[58] J. M. Morse , “A Review Committee's Guide for Evaluating Qualitative Proposals,” Qualitative Health Research 13, no. 6 (2003): 833–851. 12891717 10.1177/1049732303013006005

[59] J. Ritchie and J. Lewis , “Generalizing From Qualitative Research,” Qualitative Research Practice: A Guide for Social Science Students and Researchers (London: Sage, 2003), 263–286.

